# Predictive ethoinformatics reveals the complex migratory behaviour of a pelagic seabird, the Manx Shearwater

**DOI:** 10.1098/rsif.2013.0279

**Published:** 2013-07-06

**Authors:** Robin Freeman, Ben Dean, Holly Kirk, Kerry Leonard, Richard A. Phillips, Chris M. Perrins, Tim Guilford

**Affiliations:** 1 Centre for Mathematics and Physics in the Life Sciences and Experimental Biology, University College London, Gower Street, London WC1E 6BT, UK; 2 Computational Ecology and Environmental Science, Microsoft Research Limited, 7 JJ Thomson Avenue, Cambridge CB3 0FB, UK; 3 Animal Behaviour Research Group, Department of Zoology, University of Oxford, Oxford OX1 3PS, UK; 4 Edward Grey Institute of Field Ornithology, Department of Zoology, University of Oxford, Oxford OX1 3PS, UK; 5 Copeland Bird Observatory, c/o 16 Birch Park, Co. Down, Bangor BT19 1RZ, UK; 6 British Antarctic Survey, Natural Environment Research Council, High Cross, Madingley Road, Cambridge CB3 0ET, UK

**Keywords:** migration, behaviour, foraging, bio-logging, machine learning, ethoinformatics

## Abstract

Understanding the behaviour of animals in the wild is fundamental to conservation efforts. Advances in bio-logging technologies have offered insights into the behaviour of animals during foraging, migration and social interaction. However, broader application of these systems has been limited by device mass, cost and longevity. Here, we use information from multiple logger types to predict individual behaviour in a highly pelagic, migratory seabird, the Manx Shearwater (*Puffinus puffinus*). Using behavioural states resolved from GPS tracking of foraging during the breeding season, we demonstrate that individual behaviours can be accurately predicted during multi-year migrations from low cost, lightweight, salt-water immersion devices. This reveals a complex pattern of migratory stopovers: some involving high proportions of foraging, and others of rest behaviour. We use this technique to examine three consecutive years of global migrations, revealing the prominence of foraging behaviour during migration and the importance of highly productive waters during migratory stopover.

## 1. Introduction

As biodiversity loss accelerates, identifying key habitats and effective protected area networks becomes increasingly important [1], but is particularly difficult for animals with elusive life histories such as many marine and migratory species [2]. Recent advances in bio-logging technology have helped, but while we are beginning to understand the patterns of migratory movement in ever smaller species using simple, lightweight devices such as geolocators, knowledge of how individual animals behave on migration remains restricted to larger species able to carry complex measuring devices (e.g. GPS, time-depth or other loggers [3]). Here, we introduce a novel approach that uses a predictive model (a neural network) trained using supervised learning on a small, high-resolution sample of combined tracking data to recognize behavioural states in a larger sample of animals tracked on migration, i.e. for much longer, using simpler, lighter devices. Our method will allow researchers to use data from heavier-weight devices (such as GPS) to infer a richer understanding of behaviour in simpler data. This *ethoinformatics* approach combines the wealth of bio-logging data that we have available with a data-driven predictive model to identify complex patterns of behaviour outside the breeding season, offering a method with broad applicability for understanding global behaviour distributions in elusive species relatively cheaply and with minimal impact.

We use this method to investigate the migratory behaviour of the Manx Shearwater (*Puffinus puffinus*), a small, pelagic, diving, trans-equatorial migrant seabird exemplifying many of the limitations faced by modern bio-logging studies of migration. We demonstrate that we can predict behavioural classifications derived from rich behavioural data (high-resolution GPS tracking) *solely* from simpler data (light/immersion logging devices). To achieve this, we initially construct a dataset of labelled behaviours from GPS data, then train a neural network to predict these behavioural classes. The performance of this prediction is then assessed on independent data, and then applied to predict behaviour in data from multi-year migrations.

Using this method, we are able to predict changes in the patterns of sustained flight, rest and foraging throughout the annual cycle. We demonstrate significant behavioural shifts between three key stages of the annual cycle: breeding, migration and wintering, and show that the migration of shearwaters is behaviourally complex, with foraging occurring throughout the migratory journey interspersed with resting stopovers and periods of flight. We then identify the location of these behaviours during migration, mapping the distribution of foraging hotspots, stopover areas and flyways. Finally, we relate these behaviours globally to underlying environmental variables.

## 2. Results

### 2.1. Behavioural classification of GPS tracks

**Figure 1.**
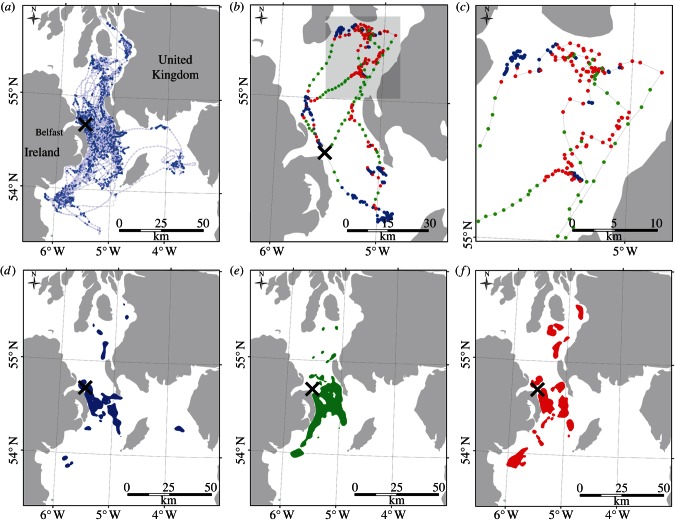
Behavioural classification of GPS trajectories for birds breeding on Copeland (marked X) in the Irish Sea. (*a*) All tracks shaded according to their corresponding salt-water immersion (darker representing more time immersed). (*b*) An example track (ET01744) classified into three behaviours: rest (blue), flight (green) and foraging (red). (*c*) Highlights the northeastern section of (*b*). (*d*–*f*) Show 50% occupancy contours for this classification applied to the entire dataset ((*d*), rest; (*e*), flight; (*f*), foraging).

Our initial labelling of behaviour from 20 GPS-tracked birds during the breeding season is shown in figure 1, using speed and tortuosity measures to classify each location (see §4). As expected, resting behaviour was commonest near the colony where birds in large social flocks (rafts) wait for nightfall to visit their burrows in safety, but periods of night-time roosting away from the colony were also seen. Flight behaviour was more diffuse, owing to birds commuting between colony and important foraging areas. Finally, foraging was concentrated both near the colony and in clusters further north and south.

### 2.2. Predicting behaviour during annual movements

**Figure 2.**
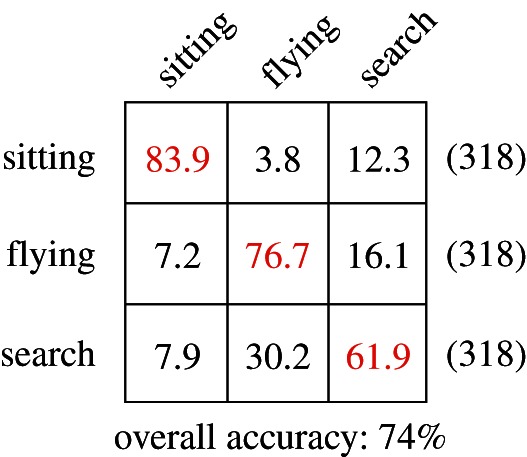
Confusion matrix for prediction performance on unseen data. Each cell shows the accuracy of classifications for a given class (row labels show the true class, and column labels show the predicted class). Correct predictions are given on the diagonal, highlighted in grey (red). (Online version in colour.)

Our method was able to predict successfully the behavioural classifications in independent data from salt-water immersion/light-level alone with an overall success of 74 per cent, which was significantly higher than expected by chance. Figure 2 shows the confusion matrix for these predictions on independent validation data.

Applying these predictions to salt-water immersion data from year-round tracking of 33 individuals carrying light-level/immersion loggers over three consecutive years (8 from 2007 to 2009, 13 from 2008 to 2009 and 12 from 2009 to 2010) showed significant differences in behavioural patterns between breeding, migration and wintering stages. There were significant effects of stage, individual and their interaction (individual: *p* < 0.001, *partial *η**^2^ = 0.03; stage: *p* < 0.001, *partial *η**^2^ = 0.18; stage × individual: *p* < 0.001, *partial *η**^2^ = 0.04; see the electronic supplementary material for full statistics). These effects were evident in multiple years, with differences remaining significant and stage having a much larger effect size than individual or the interaction term (see the electronic supplementary material, table S1). The percentage of time (median, 25th–75th centile) spent foraging during the breeding season varied, but was generally greater (11%, 0.04–0.18) than in winter (7%, 0.01–0.13). The percentage of time spent foraging was higher still during migration (14%, 0.07–0.24), but more variable. The percentage of time engaged in flight behaviour was lower in winter (1%, 0.0–0.06) than during breeding (8%, 0.01–0.19) or migration (24%, 0.08–0.39). This reduction in foraging and flight during winter was accompanied by a corresponding increase in rest behaviour during the winter (90%, 0.81–0.99) compared with during breeding (75%, 0.61–0.89) or migration (60%, 0.42–0.74).

**Figure 3.**
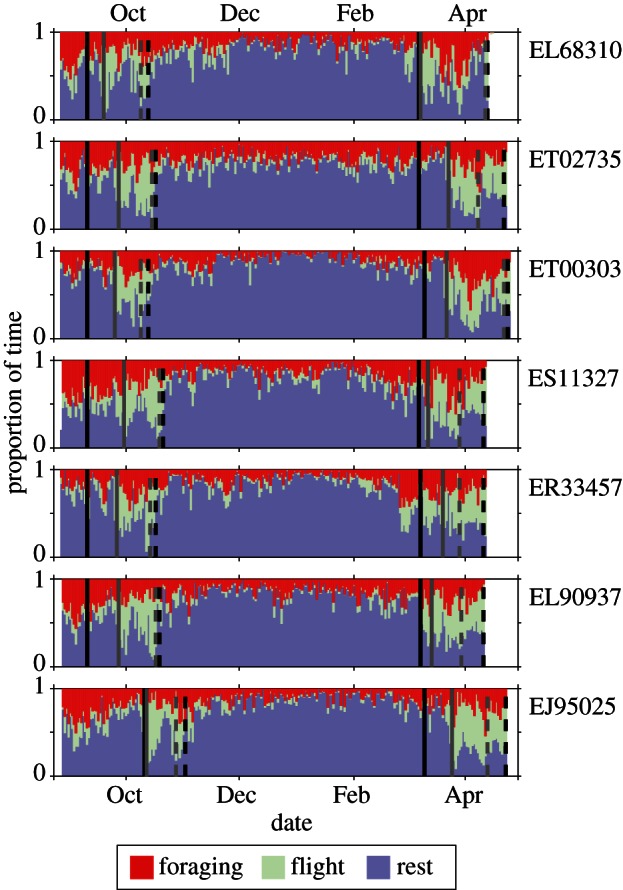
Proportion of each day spent in each of three behavioural states for a sample of individuals tracked during 2007–2008. Vertical black lines indicate when the individuals were first (dotted) and last (solid) recorded within 1200 km of the colony or median wintering location. Vertical grey lines indicate the start and end of significant flight bouts as determined by salt-water immersion (see the electronic supplementary material).

Each stage is composed of a complex time series of behaviour for each bird (see figure 3 and electronic supplementary material, figures S1–S3). A general increase in rest and corresponding decrease in foraging behaviour was evident during winter. We also noted variation in the proportion of foraging behaviour within each stage (figure 3, red). To examine this, we divided each of the breeding and wintering stages into three sub-stages, comprising the first and last 30 days, and middle of each stage. Although there did not appear to be an overall difference in behavioural strategy among these sub-stages (sub-stage: *p =* 0.273), *individual* behavioural strategies remained significantly different (individual: *p* < 0.001), and this interacted significantly with sub-stage (sub-stage × individual: *p* < 0.001).

### 2.3. Spatial distribution of behaviour

**Figure 4.**
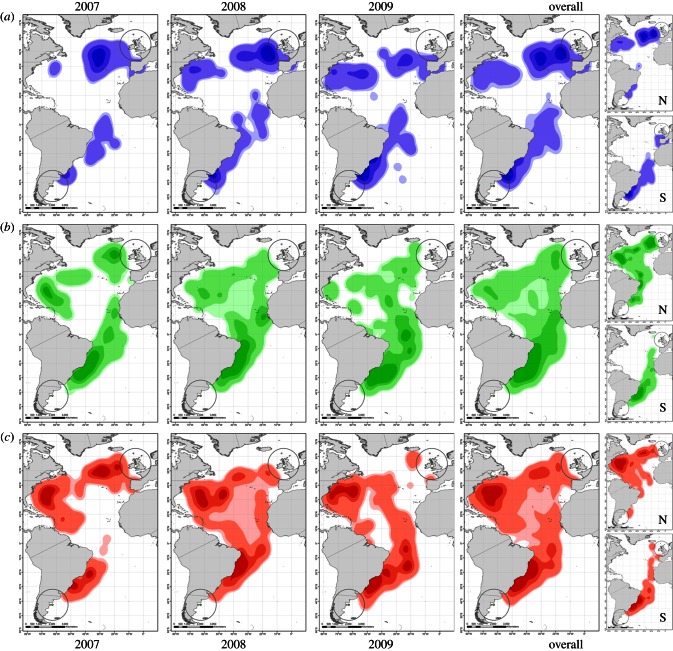
Occupancy contours for behaviours during migration. Rows (*a*–*c*) show contours for the subset of data containing the top quarter of locations ranked according to each behaviour ((*a*), rest; (*b*), flight; (*c*), foraging). Columns show yearly, then overall distributions with corresponding northbound (top) and southbound (bottom) components. Data within 1200 km of breeding colony or mid-wintering location (circles) are excluded.

Individuals engaged in high proportions of foraging and rest throughout migration (figure 4). Locations with a high proportion of rest behaviour (stopovers) were more tightly clustered than flight behaviours and were more evident near the start or end of migration. However, locations with high proportions of foraging behaviour were more diffuse, being almost as evenly distributed along the migratory route as were periods of flight. We note three discrete areas of clustered foraging behaviour: (i) the western Atlantic, and (ii) the northeastern Atlantic, during northbound migrations, and (iii) off the south-eastern coast of Brazil during southbound migrations. Over 3 years, migratory resting locations were consistently clustered near the core summer and winter areas, often preceded by high levels of flight and foraging activity. Use of the western Atlantic foraging hotspot was consistent in all 3 years, but foraging visits to the north Atlantic hotspot appear to have declined over the period of observation.

### 2.4. Environmental correlates of behaviour

**Figure 5.**
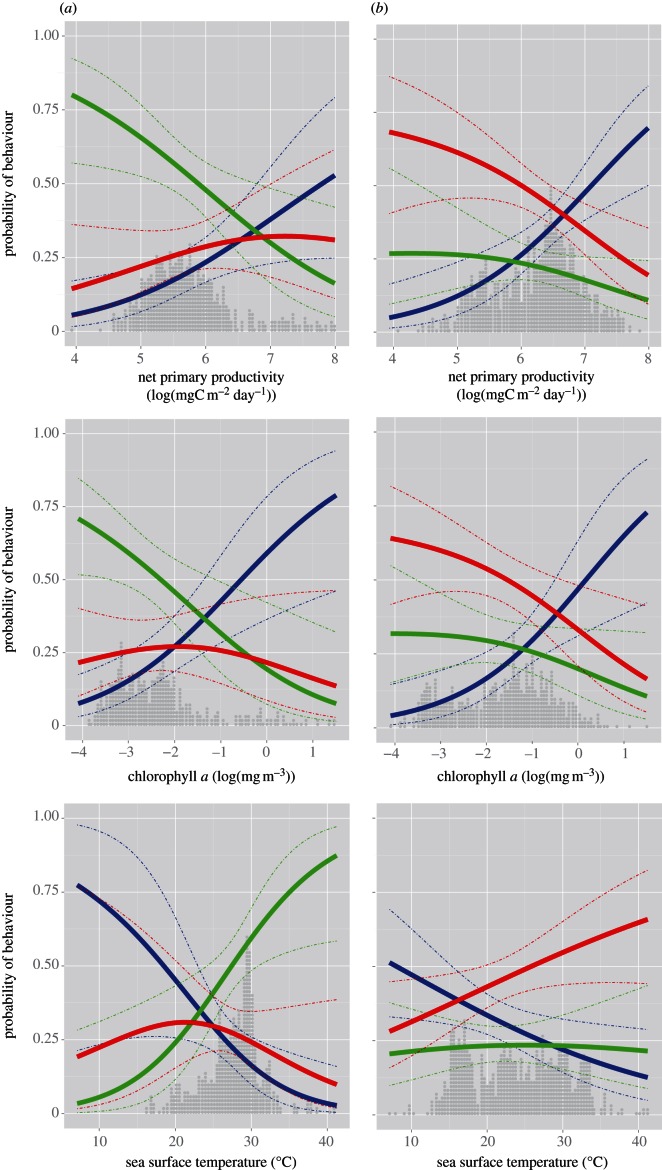
Modelled relationship between environmental variables (NPP, CHL-a and SST) and behaviour (rest, blue; flight, green; foraging, red) using multinomial logistic regression. Columns show southbound (*a*) and northbound (*b*) migrations. Solid lines show probability of particular behaviours, dotted lines show 95% CI and overlaid grey dot-plot shows distribution of individual samples across the relevant environmental range for each comparison (where each dot shows a single sample so height shows relative frequency). Axes are normalized to have equal range (e.g. there are no samples less than 15°C in the southbound migration).

Figure 5 shows the changing likelihood of the three behaviours with respect to key environmental variables at locations during migration (see the electronic supplementary material for details on environmental data sources, processing and statistical tables). Net primary productivity (NPP) had a significant effect on behaviour on both migratory directions (*p* < 0.001). Rest behaviour was more likely in locations with higher NPP, with corresponding reductions in the likelihood of foraging or flight behaviour. Foraging behaviour initially became more likely with increased productivity, but then declined, giving way to rest behaviour. Similarly, chlorophyll *a* had a significant influence on behaviour (*p* < 0.001). As chlorophyll *a* increased, rest behaviour became more likely. Furthermore, over the 3 years of the study, we observed a shift in the response of foraging behaviours to chlorophyll *a* with foraging behaviours appearing less likely in more recent years (2008) during northbound migration. Sea surface temperature (SST) also had a significant influence on behaviour (*p* < 0.05). As sea surface temperature increased, rest behaviour became less likely (*p* < 0.01). This relationship also changed over time, with foraging behaviours appearing less likely in later years (2008). During the northbound migration, overall SST at recorded locations also increased across years (2007–2008, 1.5°; 2008–2009, 1.78°; *p* < 0.01), but remained similar during southbound migrations.

During both northbound and southbound migrations, the distribution of each variable (NPP, SST and CHL-a) at locations where each behaviour was recorded differed significantly from the distribution at locations sampled randomly from the 95 per cent occupancy contour for that behaviour (*p <* 0.05, two-sample Kolmogorov–Smirnov test, see the electronic supplementary material, table S8 for details).

## 3. Discussion

Current methods for remote monitoring of individual behaviour often require complex, heavy, power-hungry transmitters or loggers, which can increase the chance of adverse impacts on study animals [4]. For example, GPS loggers can generate very spatially precise data allowing detailed analysis of individual behaviour [5]. However, the relatively high-power consumption of this technology means that compromises must be made with regard to size, longevity or frequency of measurement of GPS loggers [6]. Conversely, technologies such as geolocation by light levels [7] allow positions to be determined with much lower spatial accuracy (approx. 200 km [8]), but the devices are smaller and longer-lived, enabling biologists to determine the migratory movements of elusive species from albatrosses [9] to small passerines [10]. However, such studies have generally focused only on the relationship between position and time. Gaining insight into behaviour during migration can be much more problematic (but see [11]).

The impact of deployed devices on behaviour is an important consideration in tracking studies. Negative impacts have been demonstrated across a range of species from tracking devices of various weights (0.6–5.5% body weight; [12]). We have recently shown that the devices we deployed here did not have appreciable effects on reproductive success during the breeding season at another colony (Skomer Island) and during a more intensive tracking programme [13]. In the context of this analysis, even if individuals engage in different behaviour at sea (e.g. more flight behaviour and longer foraging trips), it is much less likely that the pattern of immersion within these behaviours will change.

Here, we draw upon our previous work [14,15] to classify the at-sea behaviour of Manx Shearwaters from GPS data collected from breeding birds into three behavioural states: rest, flight and foraging. We trained a neural network model to predict these states from salt-water immersion and light-level data, and apply these predictions to year-round data to present a previously elusive view of the global distribution of different behaviours for a migratory species. We show that at-sea behavioural distributions varied across the migratory cycle (see figure 3 and electronic supplementary material, figures S1–S3). During winter, time spent in both flight and foraging was dramatically lower than during the breeding season, and almost the entire period in the Southern Hemisphere was spent mainly at rest. This probably reflects release from the demands of reproduction (provisioning, commuting from the nest to forage), and perhaps also the increased costs of flight during winter moult.

The significant interaction between winter sub-stage suggests that winter foraging intensity is U-shaped (figure 3) for some birds with intense periods of foraging during a post-migration recovery period and preparation for their return to their northern breeding grounds. Potentially, this could be used in the future to analyse the sequence of energetic expenditure and recovery through the migratory cycle and, possibly, to investigate individual carry-over effects across years. The spatial distribution of behaviours (figure 4) reveals the complex nature of pelagic migration. Days with a high proportion of foraging behaviour occur throughout the migratory journey, with concentrations off southeastern Brazil during the southbound journey, and in the western Atlantic during the return. Rest behaviour also occurs throughout the migration, but the pattern is different, with greater concentrations towards the very end of the route in both directions. This may reflect distinct stopover types, with foraging stopovers to exploit regions of high prey availability, and rest stopovers to recover from long flight periods, or to wait until conditions for travel or foraging improve. Alternatively, these locations could arise as a consequence of moulting when individuals will remain on the sea surface for extended periods. Appropriate at-sea conservation measures are likely to differ between different stopover types.

The significant difference between the distribution of environmental variables in recorded, compared with randomized, locations indicates that birds are responding to environmental conditions. This is confirmed by the significant relationships between behaviour and environmental variables such as NPP, chlorophyll *a* and SST (figure 5). These environmental parameters covary strongly, with the measures of productivity (NPP) and chlorophyll *a* being highest in colder waters. Rest behaviour appears to be more likely in cooler and more productive waters. The changing likelihood of foraging behaviour appears more complex: initially increasing in cooler, more productive waters and then declining. This may result from the dynamics of searching and resting behaviour, with searches for prey tending to take place in less productive environmental conditions and individuals being more likely to settle in areas of high productivity. Some individuals may also be adopting a sit-and-wait strategy, remaining in productive patches to maximize their chances of encountering prey. Finding and settling in areas of high productivity could be particularly important during moulting, when flight is more energetically costly. We also noted an increase in SST at recorded locations during the northbound return in the 3 study years, coinciding with a major westwards shift in foraging behaviour from the north Atlantic hotspot towards warmer waters in the Caribbean (figure 4). In combination with the consistent targeting of highly productive waters, this suggests the capacity for a rapid response to changing oceanic conditions.

Our ethoinformatics approach may have very general applicability to existing datasets. The key is to find some factor that can be recorded cheaply (e.g. acceleration, light and immersion) and varies with behaviour and to combine this with a sub-sample of richer data (GPS, traditional field observation) in order to classify behaviour. Supervised learning (e.g. a neural network) can then be used to recognize behavioural classes from the richer data using only the information in the cheaper dataset. As the explosion of bio-logging studies continues, the development and application of methods such as ours, which allow for low-impact analysis and discovery of otherwise hidden behavioural patterns in elusive species or difficult environments, are likely to become increasingly fruitful.

## 4. Material and methods

Data were collected from Manx Shearwaters breeding on Lighthouse Island, Copeland Islands, Northern Ireland, UK (latitude 54.67° N, longitude −5.52° E), and have not been previously published. Geolocation devices, each including a salt-water immersion logger, were deployed and recovered over the course of 4 years resulting in three consecutive periods of migratory behaviour (2007/2008, 2008/2009, 2009/2010—referred to in the text by deployment year: 2007, 2008 and 2009). GPS devices were deployed during the breeding season in 2008. Study burrow entrances were marked with pegs to determine entry/exit, and regularly inspected at night to determine the possible return of adults to feed their chick. When marker pegs were disturbed, burrows were checked for feeding sounds and if an adult was present it was left until feeding was complete. The adult was then removed and fitted with a light-level geolocator/salt-water immersion logger (British Antarctic Survey Mk-14 and Mk-19 devices, weighing 1.8 and 2.5 g, respectively) and customized GPS loggers (modified igot-u GT-120s, Mobile Action, approx. 14–15 g). Geolocators were affixed to Darvic leg bands with two cable-ties (Panduit Pan-ty PLT.6SM-C0) and superglue. GPS loggers were configured to record geographical location every 5 min, then environmentally sealed in heat-shrink tubing (Finishrink CLR-20/50) and attached to four or five small bunches of back feathers with 1–1.5 cm wide strips of Tesa Tape. All-inclusive mass of devices and attachments was less than 17 g (3.5–4.5% body mass).

After a maximum of three trips, 7 days or once the GPS attachment was no longer secure, GPS devices were removed by peeling off the tapes. GPS removal took less than 10 min for each individual. Geolocation devices were downloaded, but left attached to record light and activity data for the subsequent migrations and non-breeding season.

In total, GPS tracks from 20 birds were collected, each with associated light-level and salt-water immersion data for one to three foraging trips, comprising 39 foraging trips, over 20 individuals for a total 19 174 data points covering 75.4 at-sea days. On each logged GPS track, each recorded location was assigned an immersion value from the nearest recorded value from the simultaneously recorded salt-water immersion data. Each bird therefore had one to three sequential foraging trips composed of GPS locations (latitude and longitude) each with an associated salt value (0–200), light value (0–64) and time. Year-round geolocator deployments resulted in a total of 31 recovered datasets with both light-level and salt-water immersion data (7 from 2007 to 2009, 12 from 2008 to 2009 and 12 from 2009 to 2010).

### 4.1. Geolocation data

To determine migration and wintering locations, light-level data were filtered to exclude noisy light–dark transitions, unrealistically short dark periods (less than 4 h) and points surrounding the spring and autumn equinoxes (±10 days). For each year's data, the elevation angle that minimized positional error on ground truth data was found and subsequently applied to the entire year's dataset (−3.5 for 2007 and 2008, −4.5 for 2009). Data where the average speed was consistently above 30 ms^−1^ (the maximum likely flight speed [14]) for 3 days were then removed. This removed the majority of erroneous locations, but any locations that remained at very high altitudes were also removed.

Significant flight bouts during migration (vertical grey lines in figure 4; electronic supplementary material, figures S1–S3) were estimated separately of behavioural predictions by finding those periods when daily proportion of salt-water immersion was first below 0.25 after the individuals were last recorded near the colony/wintering-grounds or before the birds were first recorded near the colony/wintering-grounds.

### 4.2. Behavioural classification

Our aim was to predict these behavioural classes derived from high-resolution GPS trajectory data during summer breeding *solely* from data available from independent geolocation–immersion devices: light and salt-water immersion data.

GPS tracks from summer foraging behaviour (19 174 data points for 20 individuals covering 75.4 at-sea days) were initially labelled into three classes delineating ‘rest’, ‘foraging’ and ‘flight’. Labelling was based on movement parameters, speed *s*: the median flight speed measured over six consecutive locations and tortuosity *t*: the arc-chord ratio over five consecutive locations. For tortuosity, the total distance is calculated between each point (arc-length) and the beeline distance from the first to the last point is also calculated (chord-length). We divide the chord-length by the arc-length giving a possible value between approximately 0 and 1, with 1 indicating a straight line and greater deviation from a straight line leading to values near 0. We find that a cut-off value of *t* = 0.98 effectively separates directed flight from undirected movement (in our data). These values were derived exclusively from GPS data. We have previously shown that there are different behavioural modes in the speed and tortuosity of flights recorded from Manx Shearwaters (at a different colony) during the breeding season [11]. Examining the GPS tracking data shows a bimodal distribution of speeds with low-speed locations (*s* < 2.5 ms^−1^: *rest*) are generally associated with resting on the surface of the water, often during the night, but also during the day when birds are rafting. High-speed, directed movements (*s* > 2.5 ms^−1^, *t* > 0.98: *flight*) are generally associated with commuting behaviours between the colony and apparent foraging and rafting locations. Finally, we observed a more complex process of high-speed, tortuous movements, which we denoted as foraging behaviour (*s* > 2.5 ms^−1^, *t* < 0.98: *foraging*). Here, the labelling of each location in the tracking data as one of these modes of behaviour was based on a combination of prior results [14] and expert observations. Such classification could also be achieved using more complex techniques (e.g. behavioural change-point analysis, [16]). Our approach gives similar results to our previous application of state-space models [13]. Critically, however, the subsequent prediction method we describe is blind to the process that creates these labels—its aim is simply to predict them.

**Figure 6.**
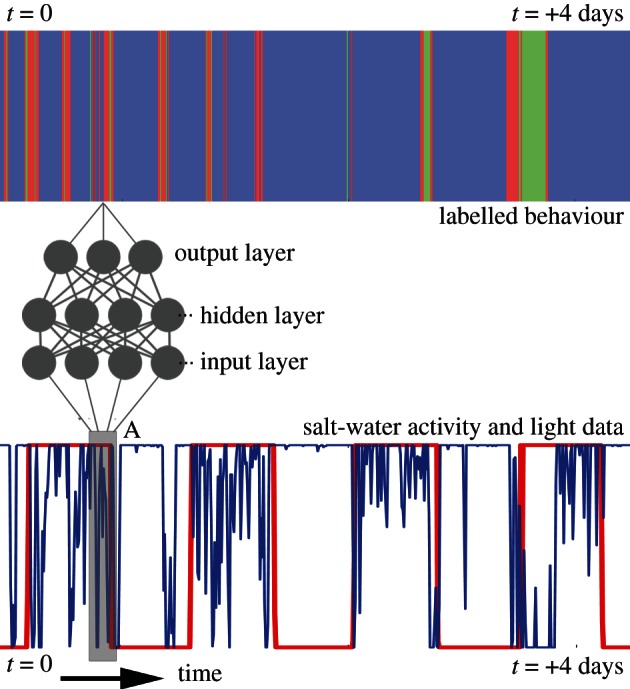
Schematic of prediction problem. Given a series of behavioural states (labelled data), we train a neural network to predict these states from a window (A) of a simpler data source (here salt-water immersion/light). Initially, class labels from a training dataset are used to train the network parameters and then the trained network is used to predict classes in novel data.

This labelled dataset was subdivided into three distinct subsets: ‘training’, ‘test’ and ‘validation’. To avoid problems associated with unbalanced data (e.g. attaining low prediction error by simply predicting the more popular behaviour), a balanced set of data was extracted with an equal number of examples (total 2000) drawn randomly from each behaviour category, then subdivided into training, test and validation subsets (50%, 25% and 25% of the total, respectively). A feed-forward neural network was trained to predict these behaviours from independently, but simultaneously, collected salt-water immersion/light-level data using the ‘training’ subset (see figure 6 for a schematic), and parameters were optimized to maximize performance on the ‘test’ subset (see the electronic supplementary material for complete network structure). Predictive performance was then assessed on the independent ‘validation’ subset and these values are given in the text (see also figure 2). This model was then used to predict behaviour on novel data from outside the breeding season from 33 individuals over 3 consecutive years. This provides a behavioural prediction for each individual every 10 min throughout the non-breeding period. We aggregate these data into days, as the proportion of each day spent engaging in the three behaviours, in order to explore the relationships between behaviour and several environmental covariates.

### 4.3. Kernel density estimations and behavioural mapping

In both the GPS tracking and geolocation data, kernel density estimation was used to extract occupancy contours for the locations. Kernel density estimations and isopleths were calculated using the Geospatial Modelling Environment (Spatial Ecology LLC). For geolocation data, kernel density estimations were calculated using a cell size of 0.1° and a bandwidth of 10°. For density estimation of GPS data (figure 1), this was reduced to a cell size of 0.01° and a bandwidth of 1°. Our aim in these figures was to convey the difference between years in these data. Rather than estimating parameters independently for each year, which would result in figures that were hard to compare, we chose to follow an established approach of selecting a consistent set of parameters over all years that produced contiguous cores without over-smoothing.

When comparing locations where individuals engaged most in a particular behaviour, the top 25 per cent of locations ranked according to each bird behaviour were extracted and their estimated densities mapped (figure 4). This could theoretically result in each period being identified as possibly in the top quarter of rest, flight or foraging, and any combination of all three. However, we found that the majority of locations in these subsets were distinct (61%), appearing only in the top quarter of a single behaviour, with 6 per cent of locations in the top quarter of two behaviours, and none in the top of all three. Thirty-three per cent of locations did not appear in the top quarter of any behaviour. Of those 6 per cent of locations that appeared in the top quarter of two behaviours, 95 per cent were composed of flight and foraging, 4 per cent were composed of rest and foraging and none were composed of rest and flight, making these distributions generally distinct.

## References

[1] Butchart SHM 2010 Global biodiversity: indicators of recent declines. Science 328, 1164–1168 10.1126/science.1187512 (doi:10.1126/science.1187512) 20430971

[2] Wood LJ Fish L Laughren J Pauly D 2008 Assessing progress towards global marine protection targets: shortfalls in information and action. Oryx 42, 340–351 10.1017/S003060530800046X (doi:10.1017/S003060530800046X)

[3] Block BA 2011 Tracking apex marine predator movements in a dynamic ocean. Nature 475, 86–90 10.1038/nature10082 (doi:10.1038/nature10082) 21697831

[4] Barron DG Brawn JD Weatherhead PJ 2010 Meta-analysis of transmitter effects on avian behaviour and ecology. Methods Ecol. Evol. 1, 180–187 10.1111/j.2041-210X.2010.00013.x (doi:10.1111/j.2041-210X.2010.00013.x)

[5] Jouventin P Weimerskirch H 1990 Satellite tracking of Wandering albatrosses. Nature 343, 746–748 10.1038/343746a0 (doi:10.1038/343746a0)

[6] Bridge ES 2011 Technology on the move: recent and forthcoming innovations for tracking migratory birds. BioScience 61, 689–698 10.1525/bio.2011.61.9.7 (doi:10.1525/bio.2011.61.9.7)

[7] Wilson RP Ducamp JJ Rees WG Culik BM Nickamp K 1992 Estimation of location: global coverage using light intensity. In Wildlife telemetry: remote monitoring and tracking of animals (eds Priede IG Swift SM), pp. 131–134 New York, NY: Ellis Horwood

[8] Phillips RA Silk JRD Croxall JP Afanasyev V Briggs DR 2004 Accuracy of geolocation estimates for flying seabirds. Mar. Ecol. Prog. Ser. 266, 265–272 10.3354/meps266265 (doi:10.3354/meps266265)

[9] Croxall JP Silk JRD Phillips RA Afanasyev V Briggs DR 2005 Global circumnavigations: tracking year-round ranges of nonbreeding albatrosses. Science 307, 249–250 10.1126/science.1106042 (doi:10.1126/science.1106042) 15653503

[10] Stutchbury BJM Tarof SA Done T Gow E Kramer PM Tautin J Fox JW Afanasyev V 2009 Tracking long-distance songbird migration by using geolocators. Science 323, 896 10.1126/science.1166664 (doi:10.1126/science.1166664) 19213909

[11] Dias MP Granadeiro JP Catry P 2012 Working the day or the night shift? Foraging schedules of Cory's shearwaters vary according to marine habitat. Mar. Ecol. Prog. Ser. 467, 245–252 10.3354/meps09966 (doi:10.3354/meps09966)

[12] Phillips Ra Xavier JC Croxall JP 2003 Effects of satellite transmitters on albatrosses and petrels. Auk 120, 1082 10.1642/0004-8038(2003)120[1082:EOSTOA]2.0.CO;2 (doi:10.1642/0004-8038(2003)120[1082:EOSTOA]2.0.CO;2)

[13] Dean B Freeman R Kirk H Leonard K Phillips RA Perrins CM Guilford T 2012 Behavioural mapping of a pelagic seabird: combining multiple sensors and a hidden Markov model reveals the distribution of at-sea behaviour. J. R. Soc. Interface 10, 20120570 10.1098/rsif.2012.0570 (doi:10.1098/rsif.2012.0570) PMC3565783 23034356

[14] Guilford TC Meade J Freeman R Biro D Evans T Bonadonna F Boyle D Roberts S Perrins CM 2008 GPS tracking of the foraging movements of manx shearwaters puffinus puffinus breeding on skomer island, wales. Ibis 150, 462–473 10.1111/j.1474-919X.2008.00805.x (doi:10.1111/j.1474-919X.2008.00805.x)

[15] Guilford T Meade J Willis J Phillips Ra Boyle D Roberts S Collett M Freeman R Perrins CM 2009 Migration and stopover in a small pelagic seabird, the Manx shearwater *Puffinus puffinus*: insights from machine learning. Proc. R. Soc. B 276, 1215–1223 10.1098/rspb.2008.1577 (doi:10.1098/rspb.2008.1577) PMC2660961 19141421

[16] Gurarie E Andrews R Laidre K 2009 A novel method for identifying behavioural changes in animal movement data. Ecol. Lett. 12, 395–408 10.1111/j.1461-0248.2009.01293.x (doi:10.1111/j.1461-0248.2009.01293.x) 19379134

